# What Shall We Do to Enter the Kingdom of Materia Medica

**Published:** 1889-02

**Authors:** S. Lilienthal


### WHAT SHALL WE DO TO ENTER THE KINGDOM OF MATERIA MEDICA?

Every earnest student of the materia medica has asked himself the same question over and over, and the thanks of all of us are due to Dr. Vandenburg, who, in the December number of The Homœopathic Physician took the bull by the horns and angered it at once by the word "concomitance;" for, though each drug has its particular symptoms, they, *taken collectively*, surely indicate the drug class! Hahnemann's arrangement absolutely destroys this relation of concomitance.

It is true, the British Homœopathic Society and the American Institute of Homœopathy have published two volumes of the *Cyclopædia of Drug Pathogenesy*, and two more volumes are promised. My criticism on the first two volumes has been severely attacked, and still I cannot retract one iota; and it is not the fault of the editors that it is only fragmentary for the microscope dictated harsh rules from which to depart the permission of the Societies must first be gained. I looked through several of the provings, as given in the pathogenesis, and I just missed the uncommon and peculiar symptoms, which, with their concomitants to this symptom, would give us the corresponding picture.

We must have a full and exhaustive *Cyclopædia of Drug Pathogenesy*, no matter whether the proving was made with the or the MM. *Such work must be paid for.* Who will open the subscription list, not for the finished work, but for the making of it? Put my name down for the first one hundred dollars. I think it should be easy to get three or four thousand dollars together for such a plan, especially as it does not interfere with the work of the Societies. With a full drug pathogenesy combined to Litten's ten volumes and Lee's promised repertory, the kingdom of the materia medica would be opened to saints and sinners.

S. LILIENTHAL.

